# Acute inflammatory profiles differ with sex and age after spinal cord injury

**DOI:** 10.1186/s12974-021-02161-8

**Published:** 2021-05-13

**Authors:** Andrew N. Stewart, John L. Lowe, Ethan P. Glaser, Caitlin A. Mott, Ryan K. Shahidehpour, Katelyn E. McFarlane, William M. Bailey, Bei Zhang, John C. Gensel

**Affiliations:** 1 Department of Physiology, Spinal Cord and Brain Injury Research Center, University of Kentucky College of Medicine, Lexington, KY 40536 USA; 2 Science Honors Program of Georgetown College, Georgetown, KY 40324 USA; 3 Department of Neuroscience, Spinal Cord and Brain Injury Research Center, University of Kentucky College of Medicine, Lexington, KY 40536 USA; 4 Present address: Brain and Mind Research Institute, Weill Cornell Medicine, New York, NY 10021 USA

**Keywords:** Acute inflammation, Aging, Gender, Macrophage phenotype, Neurotrauma, P2y12 receptor, Sex as a biological variable

## Abstract

**Background:**

Sex and age are emerging as influential variables that affect spinal cord injury (SCI) recovery. Despite a changing demographic towards older age at the time of SCI, the effects of sex or age on inflammation remain to be elucidated. This study determined the sex- and age-dependency of the innate immune response acutely after SCI.

**Methods:**

Male and female mice of ages 4- and 14-month-old received T9 contusion SCI and the proportion of microglia, monocyte-derived macrophages (MDM), and neutrophils surrounding the lesion were determined at 3- and 7-day post-injury (DPI) using flow cytometry. Cell counts of microglia and MDMs were obtained using immunohistochemistry to verify flow cytometry results at 3-DPI. Microglia and MDMs were separately isolated using fluorescence-activated cell sorting (FACS) at 3-day post-injury (DPI) to assess RNA expression of 27 genes associated with activation, redox, and debris metabolism/clearance.

**Results:**

Flow cytometry revealed that being female and older at the time of injury significantly increased MDMs relative to other phagocytes, specifically increasing the ratio of MDMs to microglia at 3-DPI. Cell counts using immunohistochemistry revealed that male mice have more total microglia within SCI lesions that can account for a lower MDM/microglia ratio. With NanoString analyses of 27 genes, only 1 was differentially expressed between sexes in MDMs; specifically, complement protein C1qa was increased in males. No genes were affected by age in MDMs. Only 2 genes were differentially regulated in microglia between sexes after controlling for false discovery rate, specifically CYBB (NOX2) as a reactive oxygen species (ROS)-associated marker as well as MRC1 (CD206), a gene associated with reparative phenotypes. Both genes were increased in female microglia. No microglial genes were differentially regulated between ages. Differences between microglia and MDMs were found in 26 of 27 genes analyzed, all expressed higher in MDMs with three exceptions. Specifically, C1qa, cPLA2, and CD86 were expressed higher in microglia.

**Conclusions:**

These findings indicate that inflammatory responses to SCI are sex-dependent at both the level of cellular recruitment and gene expression.

**Supplementary Information:**

The online version contains supplementary material available at 10.1186/s12974-021-02161-8.

## Introduction

Inflammation occurring after spinal cord injury (SCI) plays a juxtaposing role by promoting both tissue repair while further exacerbating tissue damage [1, 2]. Innate immune cells including neutrophils and monocyte-derived macrophages (MDM) that infiltrate the damaged spinal cord, along with activated resident microglia, aid in clearing cellular debris [3, 4]. Cytokines, growth factors, enzymes, and proteases released by inflammatory leukocytes and microglia participate in tissue remodeling that encourages axon sprouting and cellular migration, which can promote both functional recovery and aberrant plasticity [2]. Similarly, myeloid cells aid in clearing cellular debris but also produce high quantities of reactive oxygen species (ROS) that further exacerbate spinal damage after injury [5].

Despite increased efforts to understand the relative contribution of different innate immune cells in the pathophysiology of SCI; it remains important to better characterize inflammatory profiles within the injured environment and delineate differences between the functional roles of MDMs and microglia acutely after SCI. Microglia function as early phagocytic responders to local spinal damage and begin clearing cellular debris, while infiltrating MDMs assume the burden of debris clearance by 3-day post-injury [3] (DPI). Delineating functional differences between MDMs and microglia after SCI is considerably challenged due to a changing spatial and temporal environment following injury. To compound this complexity, emerging evidence also suggests that physiologic variables such as sex or age at the time of neurotrauma can change inflammatory responses [6–9]. Whether similar interactions exist following SCI is understudied.

This study aimed to determine if sex and age at the time of SCI affect the inflammatory response of MDMs and microglia acutely following injury. Fluorescence-activated cell sorting (FACS) and immunohistochemistry were used to characterize the inflammatory profiles of infiltrating MDMs and neutrophils, as well as resident microglia. MDMs and microglia were collected at 3-DPI and analyzed for the expression of 27 genes associated with macrophage activation, reactive oxygen species (ROS) production, and debris clearance. Flow cytometry revealed an increased proportion of infiltrating MDMs relative to microglia and neutrophils at 3-DPI in females, which is consistent with a larger microglial response in males detected using immunohistochemistry. Gene expression analysis indicated that most genes associated with conventionally defined M1 and M2 phenotypes, as well as genes associated with ROS, were increased at higher levels in MDMs compared to microglia. However, CD86, the complement protein C1qa, and cPLA2 were increased in microglia. A sex-dependent increase in C1qa was also found in male MDMs, and an increase in the gene encoding NADPH-Oxidase (NOX2), CYBB, as well as M2-associated gene MRC1, were increased in female microglia. Collective data derived from this study implicate sex and age as differential regulators of inflammation after SCI.

## Methods

### Spinal cord injury

All procedures are approved by the University of Kentucky’s Institutional Animal Care and Use Committee. In total, 74 female and male C57Bl/6 mice of ages 4- (*n*=33; Jackson Laboratories), and 14-month old (MO; *n*=41; derived from Jackson Laboratory but aged by the National Institute on Aging) received laminectomy only as a sham injury, or laminectomy with 60- or 75-kDyn SCI under ketamine (100.0 mg/kg) and xylazine (10.0 mg/kg) anesthesia as previously described using the Infinite Horizons Impactor (Precision Systems Instrumentation, LLC, Fairfax Station, VA) [10]. After SCI, all mice received buprenorphine (Buprenex SR, 1.0 mg/kg day of surgery), as well as an antibiotic (Enrofloxacin, 5.0 mg/kg) dissolved in saline (1.0 mL/day) for up to 5 days post-SCI. Manual bladder expressions were performed 2x/day for the duration of the study. Mice of ages 4- and 14-MO were chosen as a representative shift in average clinical demographics from 27 to 43 years old at the time of SCI since the 1970s [11].

### Flow cytometry

#### Myeloid cell isolation

Mice receiving 75 kDyn SCI were anesthetized at 3- and 7-DPI using an overdose of ketamine and xylazine and were perfused using sterile 0.01 M phosphate-buffered saline (PBS) containing 1.0 mM EDTA. An 8.0-mm section of the spinal cord surrounding the SCI lesion was dissected and placed in a digestion buffer containing a 1:1 solution of RPMI and Accumax (Thermo Fisher Scientific, Waltham, MA). The cords were mashed through a 70-μm diameter meshing into a 30% Percoll solution diluted in RPMI (Thermo Fisher Scientific, Waltham, MA). Cells were centrifuged at 1500 G for 15 min at 10^o^C to pellet cells, and the floating myelin layer was aspirated. Myeloid cells and debris were then suspended in red blood cell lysis buffer and pelleted. Cells were washed in PBS and suspended in cell staining buffer (Cat #: 420201; Biolegend, San Diego, CA) for antibody labeling.

In total, *n*=35 mice were used for flow cytometry experiments split between 3- (*n*=23) and 7-DPI (*n*=12). For the 3-DPI experiments, female (*n*=3, 4-MO and *n*=5, 14-MO) and male (*n*=3, 4-MO and *n*=6, 14-MO) mice survived until perfusions and were used for flow cytometry experiments. For the 7-DPI experiments, female (*n*=3, 4- and 14-MO) and male (*n*=3, 4-MO; and *n*=2 14-MO) mice survived until perfusions. There was a loss of 4- (*n*=1) and 14-MO (*n*=2) females, as well as 14-MO (*n*=3) males which died within the first 72-h for the 3-DPI experiment, and *n*=1, 14-MO male that died at 4-DPI in the 7-DPI experiment. The imbalance in final animal numbers between groups is due to increased 14-MO animals being initiated in the experiment to compensate for a higher expected mortality. Consequently, more older mice ended up in the 3-DPI experiment despite this increased mortality. Sham mice were not performed because the uninjured spinal cords do not contain neutrophils or MDMs.

#### Antibody labeling and FACS

Endogenous Fc receptors were blocked for 15 min on ice using neutralizing antibodies targeting CD16/32 (1:100; Fc Block; Cat #: 553142; BD Biosciences, San Jose, CA). Next, cells obtained from the spinal cord were washed and resuspended in an antibody cocktail labeling all cells of the myeloid and lymphocyte lineage (Rat anti-CD45-APC conjugate; 1:100; Cat #: 559864; BD Biosciences, San Jose, CA), as well as for the macrophage-specific integrin marker (Rat anti-CD11b-PE conjugate; 1:200; Cat #: 553311; BD Biosciences, San Jose, CA) and a neutrophil selection marker (Rat anti-Ly6G-PE-Cy7 conjugate; 1:50; Cat #: 560601; BD Biosciences, San Jose, CA). Antibodies were incubated for 1 h on ice before washing and re-suspending in cell staining buffer.

**Fig. 1:**
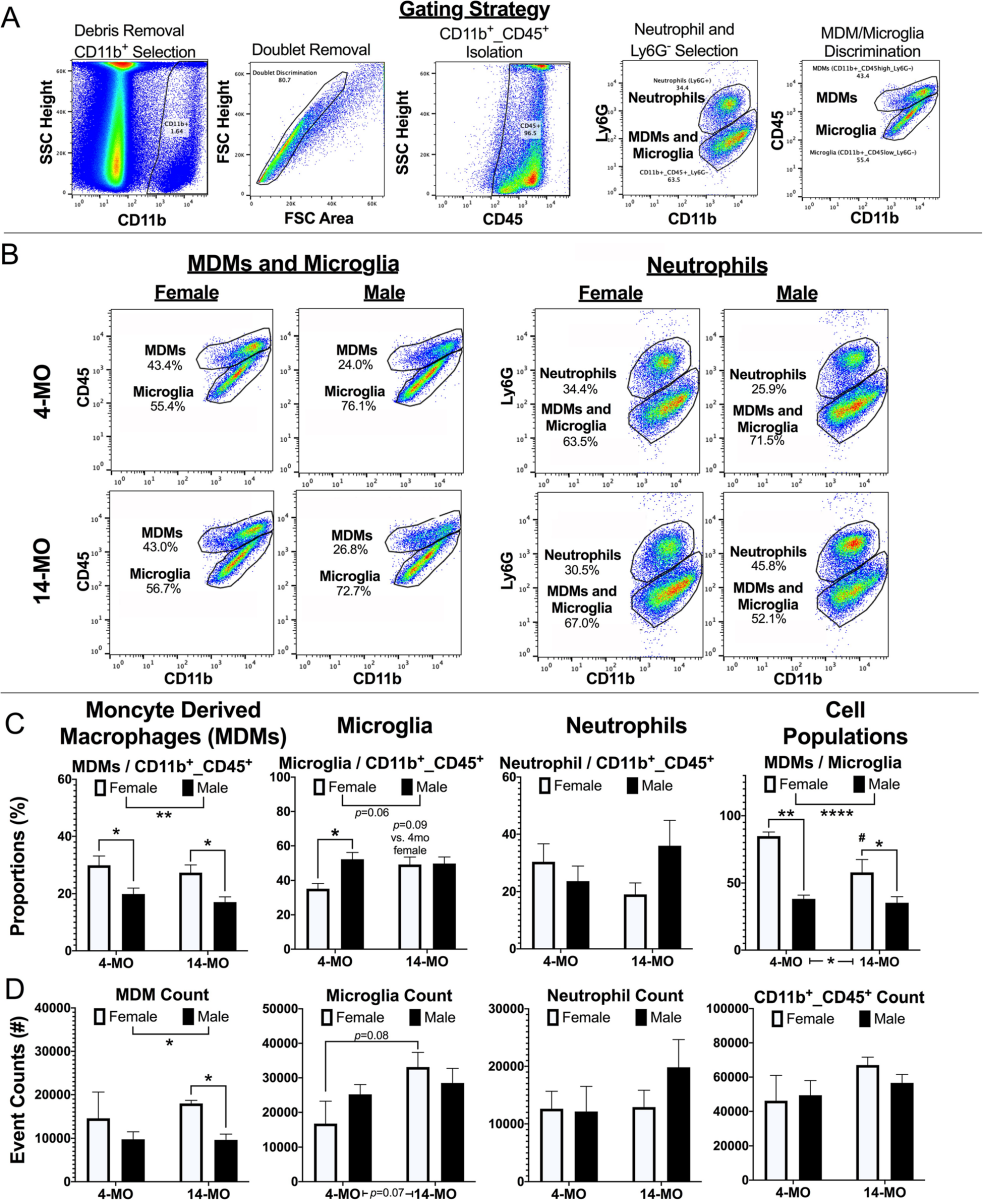
Flow cytometry reveals different inflammatory profiles between sexes at 3-DPI. **a** Myeloid cells were isolated from the injured spinal cords of male and female, 4- and 14-MO mice at 3-DPI using a consistent gating strategy. **b** Representative flow plots of cells that were immuno-labeled for selection markers against all myeloid cells (CD11b and CD45) and neutrophils (Ly6G). **c** Female mice presented with a larger proportion of MDMs compared to both microglia (*p*<0.0001), and total myeloid cells (*p*<0.01), with a main effect of sex for total MDM events (*p*<0.05). **c** Male mice presented with trends towards a larger proportion of microglia to total myeloid cells (*p*=0.053). Sample size include: 4-MO female (*n*=3), 4-MO male (*n*=3), 14-MO female (*n*=4), 14-MO male (*n*=6). Main effects were assessed using two-way ANOVA, followed by pairwise comparisons using Sidak’s for post hoc analysis when appropriate. All graphs present the standard error of measurement (SEM). *p<0.05, **p<0.01

MDMs and microglia were sorted and collected in separate tubes containing cell lysis buffer (Cat #: 51800; Norgen Biotek; Ontario, CAN) using FACS (iCyt sy3200; Sony Biotechnologies; San Jose, CA). FACS was performed by the University of Kentucky’s Flow Cytometry and Immune Monitoring Facility. An appropriate gating strategy was applied based on forward and side scatter plots to isolate cells from debris, exclude doublets, and set compensations using single-antibody controls (Fig. 1a). Experimental controls consisted of no-stain, single-channel, and fluorescence minus one. Cells obtained from 4-MO female mice that were designated for control purposes were used to set compensations. MDMs (CD45^+^/CD11b^+high^/Ly6G^-^) and microglia (CD45^+^/CD11b^+low^/Ly6G^-^) were identified and sorted into separate tubes. Neutrophils (CD45^+^/CD11b^+^/Ly6G^+^) were identified in flow plots and used for cell profiling.

Myeloid cells isolated from injured spinal cords were analyzed for the proportion of MDMs, microglia, and neutrophils to all CD45^+^/CD11b^+^ cells. Similarly, MDM/microglia ratios were used to compare total macrophage profiles. Because tubes were emptied during FACS analysis, we also used absolute cell counts to predict total inflammation and followed up on findings using immunohistochemistry to confirm results. Flow cytometry analysis was performed using Flowjo (v. 10.6.2; Ashland, OR).

#### Gene expression using NanoString

MDM and microglial RNA was collected and purified using a commercially available single-cell RNA purification kit (Norgen Biotek; Cat #: 51800; Thorold, ON, CA), and obtained RNA was quantified using Nanodrop (Nanodrop Lite; Thermo Fisher Waltham, MA) and tested for degradation using BioAnalyzer (Agilent Technologies; Santa Clara, CA). Because of the limited collection size of cells, between 4.0 and 15.0×10^3^ cells/tube/cord, RNA was eluted in 13.0 μL RNAse free-H_2_O. Only samples containing sufficient purity and a quantity of at least 25.0 ng at 2.5 ng/μL were used for RNA analysis, RNA analysis was performed using Nanostring nCounter (nCounter SPRINT profiler; NanoString Technologies; Seattle, WA), available at the University of Kentucky’s Genomics Core Laboratory. A custom code-set was created and purchased to identify genes that our lab has previously associated with macrophage activation and phenotype using Taqman gene array, as well as functional genes associated with ROS and myelin processing (a full list of RNA targets can be found in Additional file 1 and is based upon previous in vitro phenotyping [12]). In total, 59 probes were used, 6 being machine-positive controls, 8 as machine-negative controls, 3 being housekeeping genes for normalization, 5 being cell-type assay controls to determine sensitivity and purity of the sorting procedure, and 37 markers of macrophage phenotype and function. Of the 37, 10 were consistently undetectable in the samples. Any gene with less than 75% of samples testing below the threshold was excluded from the analysis. Of the 27 remaining detectable phenotypic/functional markers, 17 were designated as markers of macrophage activation, 8 were designated as markers of ROS, and 2 were designated markers of debris metabolism/clearance. NanoString nCounter was performed according to the manufacturer’s recommendations. Data was analyzed using Nanostring’s nSolver software, normalizing values to the geometric means of the housekeeping genes. A threshold was set to 20.0, or approximately 2x the geometric mean of the negative controls (geomean=10.1).

All mice used for flow cytometry experiments at 3-DPI were utilized for NanoString analysis except *n*=1, 14-MO male mouse due to degraded RNA, as well as *n*=1 microglia sample from a 14-MO female mouse being below the 25-ng limit. Mice within each variable were collapsed across the opposing variable for analyses. For example, determining effects of sex resulted in the grouping 4- (*n*=3) and 14-MO (*n*=4 for MDMs, *n*=3 for microglia) females (*n*=7 and 6 in total for female MDMs and microglia), as well as 4- (*n*=3) and 14-MO (*n*=5) males (*n*=8 total for male MDMs and microglia). For determining effects of age, 4-MO males and females (*n*=6 total for each sex for both MDMs and microglia), and 14-MO males and females (*n*=9 for MDMs and *n*=8 for microglia) were grouped together. For comparing NanoString profiles between MDMs (*n*=15) and microglia (*n*=14), all samples were grouped from both sexes and ages.

### Immunohistochemistry

Immunohistochemistry was performed on tissue obtained at 3-DPI. The injury severity was reduced to 60 kDyn due to complications with early mortality of 14-MO male mice at this injury severity (33.3% of our starting sample) as previously discussed [13]. Briefly, 14-MO male mice were dying overnight or becoming moribund within a few days post-SCI which led to challenges obtaining a sufficient sample size for flow cytometry by 7-DPI. To limit this mortality, the lesion severity was reduced from 75- to 60-kDyn. No early mortality occurred for this immunohistochemistry experiment. In total, 40 mice were used for this experiment split between mice receiving sham (*n*=3/group) or SCI (*n*=7/group). One 4-MO female sample receiving SCI was lost due to damage during tissue processing.

Mice were anesthetized using an overdose of ketamine and xylazine as described above. Mice were perfused using cardiac puncture first with 0.1 M PBS, then with 4% formaldehyde in PBS made from paraformaldehyde. The spinal cords were extracted from mice, then post-fixed for 2 h at room temperature and washed in 0.2 M phosphate buffer (PB) overnight. The cords were transferred to a 30% sucrose solution for 1 week before blocking in optimal cutting temperature compound (OCT; Thomas Scientific; Swedesboro, NJ). The spinal cords were blocked in a random order but ensuring a representation of all groups in each block (*n*=5/block). Blocks were cut between −18^o^C and -20^o^C at 10.0-μm thick in the coronal plane. Each block was cut in a series of 10 equal representative sets with 100.0 μm of *z*-distance between repeated sections. First, one set was stained as previously described [8] using eriochrome cyanine and neurofilament (Chicken anti-NF-200; 1:1,500; Cat #: NFH; Aves Laboratories; Davis, CA) to identify spared tissue. The section containing the least amount of spared tissue was considered the lesion epicenter and was used to standardize analysis with respect to this objectively defined landmark as established previously [14]. The next set was labeled for a pan macrophage marker (Rat anti-F4/80; 1:250; Cat #: MCA497; Bio-Rad AbD Serotec; Oxford, UK), a microglial specific marker (Rabbit (Rb) anti-P2y12; 1;1,000; Cat #: AS-55043A; Anaspec Inc; Fremont, CA), and with cresyl violet for nuclear detection. Microglial Rb anti-P2y12 was first labeled using a horseradish peroxidase (HRP) conjugated goat (Gt) anti-Rb secondary antibody (Cat #: PI-1000; Vector Labs; Burlingame, CA). P2y12 receptor was detected using 3,3’-diaminobenzidine (DAB) with nickel to label microglia black. Next, peroxidase from the first round of labeling was quenched using 3.0% H_2_O_2_ in 0.1 M PBS for 30 min at room temperature. An HRP conjugated Gt anti-Rat secondary antibody (mouse absorbed; Cat #: PI-9401; Vector Labs; Burlingame, CA) was then bound to the Rat anti-F4/80 antibody, followed by development using DAB without a nickel to produce a contrasting brown label in all macrophages. The tissue was then counter-stained in cresyl violet for nuclear detection, then dehydrated using graded ethanol baths and cleared using Histoclear (VWR Scientific; Radnor, PA). Slides were coverslipped with Permount (Fisher Scientific; Waltham, MA) and imaged at 20x using Axioscan (Axioscan Z1; Carl Zeiss AG; Oberkochen, DE).

Slides were visualized and analyzed using Halo software (Indica Labs; Albuquerque, NM). In total, 3 sections were used to analyze cell counts of MDMs and microglia at the lesion epicenter as well as at 100-μm rostral and caudal to the epicenter. The region of analysis was limited to within the lesion, and cells were counted using unbiased procedures adapted from fluorescent labeling techniques [8]. Annotations encompassing the lesion were partitioned into evenly spaced, but randomly placed, 100-μm^2^ boxes. MDMs were identified by the presence of brown labeling (F4/80) and all cells containing a nucleus that were within the box, or touching the top or right margins, were counted. Microglia were identified by the presence of black P2y12 labeling which often masked nuclear cresyl violet staining; therefore, a standard criterion was set for counting microglia. All P2y12 labeling that presented with a soma and at least 5 μm of continual P2y12 staining, either in diameter or length, were counted. Staining length was assessed using a built-in ruler tool in Halo. Due to differences in sizes between 4- and 14-MO mouse spinal cords, as well as occasional rips and folds in tissue, cell counts were normalized to the total area measured to obtain a cell density that is comparable between groups (examples of annotation markups and sampling procedures can be found in Supplementary Figure 1). Cells were counted by an experimenter blinded to group identities.

### Statistics

#### Flow cytometry and immunohistochemistry

Two-way analysis of variance (ANOVA) was used to test the main effects of sex and age as well as for interactions. Sidak’s pairwise comparisons were used as post hoc analysis to test effects between groups when the main effect was found significant at *p ≤* 0.05. All stats for flow cytometry and immunohistochemistry were performed using Prism (v. 8.2.1; GraphPad Software, Inc.; San Diego; CA).

#### NanoString gene expression

A general linear model was constructed for a two-way multiple analysis of variance (MANOVA) to test the main effects of sex and age as well as for interactions. Overall effects and main effects of each gene were determined. Estimated marginal means (EMM) and corresponding standard error of measurements were derived from MANOVA corrections to control for the grouped variable; i.e., correcting for the age when testing for sex or correcting for sex when testing for age. EMMs and derived standard errors were used for multiple *T* test to determine the significance of each gene. Significant *P* values are reported separately, along with *Q* values obtained after controlling for false discovery rate (FDR) using the two-stage step-up method of Benjamini, Krieger, and Yekutieli. A significant *Q* value was set to 5% to distinguish a true discovery. MANOVA’s were performed using SPSS (v. 25; IBM; Armonk, NY), and multiple *T* tests with FDR corrections were performed using Prism (v. 8.2.1; GraphPad Software, Inc., San Diego, CA).

## Results

### Flow cytometry reveals higher quantities and ratios of MDMs in injured spinal cords of female mice

We previously identified both sex and age as important biological variables in the pathophysiology of SCI inflammation and recovery [6, 14, 15]. Further, we identified that MDMs and microglia are the main producers of ROS in the acutely injured spinal cord and that increased ROS production contributes to age-dependent functional recovery [7, 8]. Here, we sought to determine the effect of sex and age on SCI-induced inflammation by examining peripheral and CNS immune responses after thoracic contusion SCI in male and female mice aged 4 or 14 months at the time of injury.

**Fig. 2:**
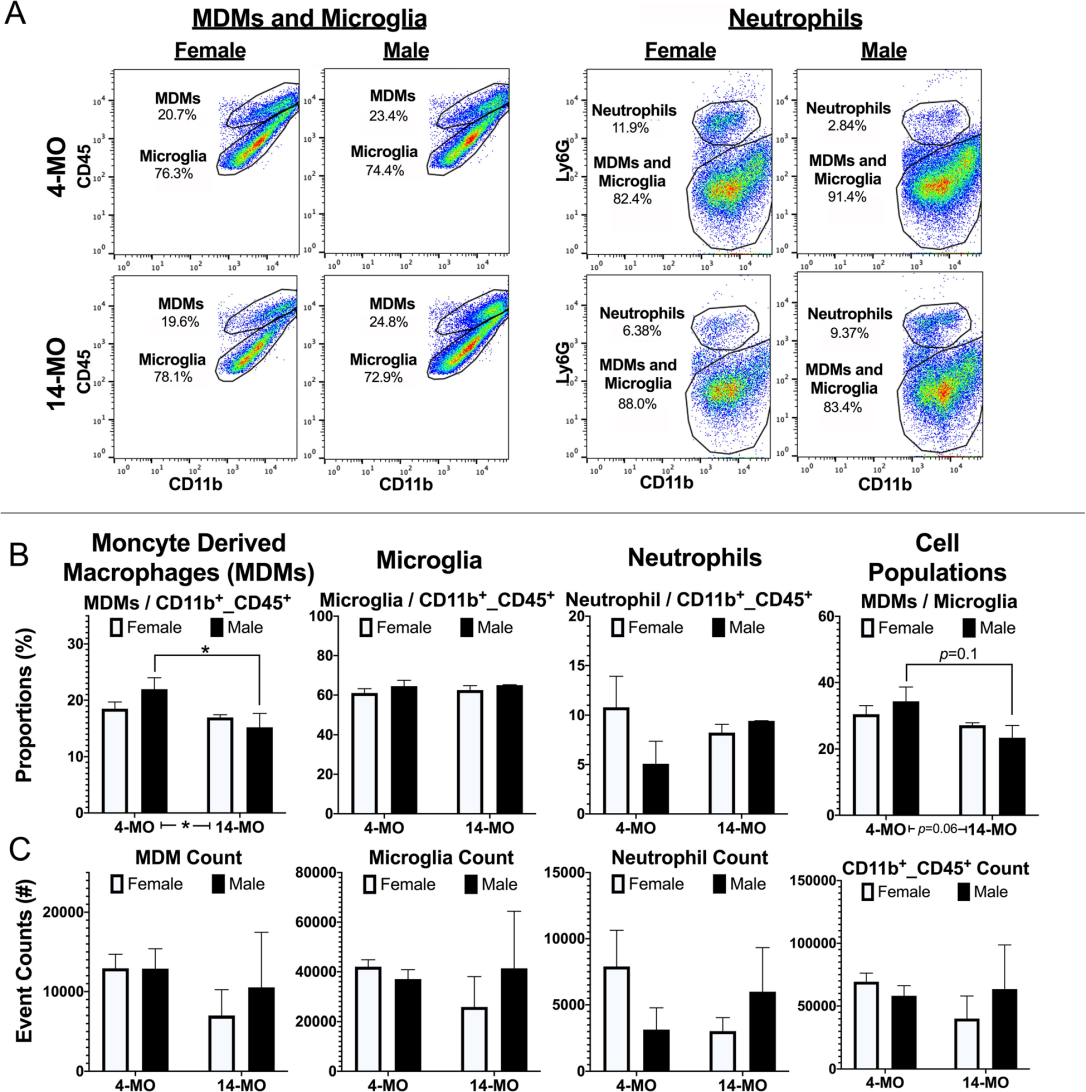
Younger mice have a higher proportion of MDMs at 7-DPI. Myeloid cells were isolated from the injured spinal cords of male and female, 4- and 14-MO mice at 7-DPI using a consistent gating strategy (Fig. 1a). **a** Representative flow plots of cells that were immuno-labeled for selection markers against all myeloid cells (CD45), phagocytic cells (CD11b), and neutrophils (Ly6G). **b** 4-MO mice presented with a larger proportion of MDMs compared to total phagocytic cells (*p*<0.05). Sample size include 4-MO female (*n*=3), 4-MO male (*n*=3), 14-MO female (*n*=3), and 14-MO male (*n*=2). Main effects were assessed using two-way ANOVA, followed by pairwise comparisons using Sidak’s for post hoc analysis when appropriate. All graphs present the standard error of measurement (SEM). *p<0.05

To determine the relative responses of MDM, microglia, and neutrophils to injury, we harvested myeloid cells from the injured spinal cord at 3- (Fig. 1b) and 7-DPI (Fig. 2a) and compared the ratios of each cell type to the overall CD45^+^/CD11b^+^ myeloid cell populations using flow cytometry. At 3-DPI, for MDMs (CD11b^+^/CD45^hi^/Ly6G^-^), we detected a significant main effect of sex (*F*_(1, 12)_=16.65, *p*<0.005; Fig. 1c). This effect was driven by both 4- (*p*<0.05) and 14-MO (*p*<0.05) female mice having a significant increase in the proportion of MDMs relative to total myeloid cells compared to age-matched males. There was no significant main effect of age (*F*_(1, 12)_=1.14, *p*=0.31) in the ratio of MDM to total myeloid cells in the spinal cord.

Next, we examined the ratio of microglia (CD11b^+^/CD45^lo^/Ly6G^-^) to total myeloid cells at 3-DPI and observed a trend for a main effect of sex (*F*_(1, 12)_=4.10, *p*=0.06; Fig. 1c) and a sex by age interaction (*F*_(1, 12)_=3.67, *p*=0.08; Fig. 1c). Both effects were driven by decreased microglia ratios in 4-MO females that reached statistical significance when compared to age-matched males (*p*=0.05) and were decreased compared to 14-MO females (*p*=0.09). There was no significant main effect of age on the ratio of microglia to total myeloid cells (*F*_(1, 12)_=1.77, *p*=0.2; Fig. 1c). The proportion of CD11b^+^/CD45^+^/Ly6G^+^ neutrophils relative to total CD11b^+^ myeloid cells was not significantly affected by sex (*F*_(1, 12)_=0.39, *p*=0.54) nor age (*F*_(1, 12)_=0.00035, *p*=0.95) at 3-DPI.

Since our results indicated that both MDMs and microglia are affected by age and sex, we next compared MDMs/microglia ratios. Our flow cytometry results indicate that being female and older at the time of injury increases MDM infiltration into the cord at 3-DPI (Fig. 1c). Specifically, there was a significant main effect of sex (*F*_(1, 12)_=29.80, *p*<0.0001; Fig. 1c) and age (*F*_(1, 12)_=5.50, *p*<0.05) in MDM/microglia ratios. Both 4- (*p*<0.001) and 14-MO (*p*<0.05) female mice had significantly higher MDMs relative to microglia ratios compared to age-matched males. The effect of age was driven by 4-MO females having a significantly larger proportion of MDMs compared to 14-MO females (*p*<0.05). There were no significant differences between 4- and 14-MO males (*p*=0.93).

Examining proportions provides insight into the inflammatory profile after injury. To better understand the magnitude of the myeloid cell response to SCI, we compared cell counts of MDMs, microglia, and neutrophils across groups by running the entirety of the prepared samples through the cytometer. No differences were found among groups for total number of myeloid cells sampled (CD45^+^/CD11b^+^; main effect of sex *F*_(1, 12)_=0.208, *p*=0.64; main effect of age *F*_(1, 12)_=3.17, *p*=0.09; Fig. 1c), thereby validating our flow cytometry proportion and count measures. For individual cell types, a significant main effect of sex emerged for MDMs (*F*_(1, 12)_=6.55, *p*<0.05; Fig. 1d), with females having higher numbers of MDMs than males. Post hoc analyses revealed a significant increase in 14-MO females compared to age-matched males (*p*<0.05). We observed a trend towards increased microglial counts with age (*F*_(1, 12)_=4.06, *p*=0.07; Fig. 1d) largely driven by increased microglia numbers in 14-MO females compared to 4-MO females (*p*=0.08). There were no significant main effects of sex (*F*_(1, 12)_=0.16, *p*=0.7; Fig. 1d) in microglia counts nor significant main effects for neutrophils in either sex (*F*_(1, 12)_=0.47, *p*=0.5) or age (*F*_(1, 12)_=0.72, *p*=0.4). Collectively, our flow cytometry data at 3-DPI demonstrates that males and females differ in their inflammatory profiles after SCI, which is driven largely by significant increases in monocytes relative to microglia in females. This occurs despite a relative potentiation of microglial activation in 14-MO females relative to younger, same-sex counterparts.

Identical analyses were performed at 7-DPI (Fig. 2). Regarding proportional analyses, we observed a significant main effect of age for MDMs compared to all myeloid (CD45^+^/CD11b^+^) cells (*F*_(1, 7)_=6.85, *p*<0.05; Fig. 2b) and post hoc analyses revealed a significant increase in 4-MO males compared to 14-MO counterparts (*p*<0.05). The proportion of microglia to myeloid cells was not significantly affected by age (*F*_(1, 7)_=0.17, *p*=0.7) no sex (*F*_(1, 7)_=1.5, *p*=0.3). Similarly, there were no significant main effects in the proportion of neutrophils to total myeloid cells with regard to age (*F*_(1, 7)_=0.16, *p*=0.7) or sex (*F*_(1, 7)_=1.02, *p*=0.3). When comparing the proportion of MDMs to microglia, we observed a trend for a main effect of age (*F*_(1, 7)_=5.15, *p*=0.06) that was driven by increases in the MDM/microglia ration in 4-MO males compared to 14-MO males (*p*=0.1). There were no significant main effects when examining cell counts (*p*=0.2) for main effects of age or sex in the total number of myeloid cells, MDMs, microglia, and neutrophils. Collectively, our 7-DPI flow analyses indicate that the early, 3-DPI, age-, and sex-dependent inflammatory outcomes largely normalized by 1-week post injury. While we did observe some potential increases in MDMs in 4-MO males at 7-DPI, it is important to note that a significant mortality in 14-MO males after SCI precluded our ability to generate significant power in this group (*n*=2) and we interpret any group differences between males with caution. We have previously reported on this aged-male-dependent mortality in a recent publication examining the effect of sex on SCI outcomes [13].

### Immunohistochemistry reveals a male-dependent increase in microglia at 3-DPI

**Fig. 3:**
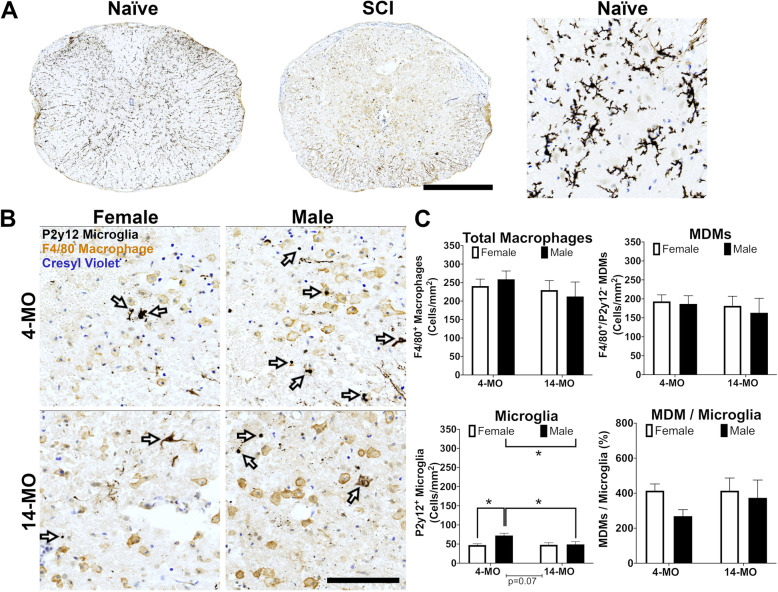
Younger male mice have a higher density of microglia within the lesion at 3-DPI. **a**, **b** Fixed tissue was obtained from male and female, 4- and 14-MO mice at 3-DPI and immunolabeled for detection of MDMs (F4/80^+^ and P2y12^-^) and microglia (F4/80^+^ and P2y12^+^). **b** Microglia cell counts presented with very heterogeneous morphology (arrows), ranging from ramified to ameboid. Monocytes are labeled consistently as large cells with a brown membrane-associated labeling. Cell counts were normalized to the total area measured. **c** Total macrophages or MDM density did not differ between groups, nor did the MDM/microglia ratios. **c** Cell count density of microglia was significantly increased in 4-MO males compared to 4-MO females (*p*<0.05*)* and 14-MO males (*p*<0.05). Sample size include: 4-MO female (*n*=6), 4-MO male (*n*=7), 14-MO female (*n*=7), and 14-MO male (*n*=7). Main effects were assessed using two-way ANOVA, followed by pairwise comparisons using Sidak’s for post hoc analysis when appropriate. All graphs present SEM. Error bars represent 500 μm (**a**) and 100 μm (**b**). *p<0.05

Flow cytometry analyses included the spinal cord tissue encompassing multiple spinal levels (8 mm) [16] including the entirety of lesion tissue. To better understand the inflammatory response at the level of the lesion epicenter, we generated tissue sections from 4- and 14-MO, male and female mice, with and without SCI (Fig. 3 a, b). To examine both cell types in situ, we sampled injured tissue at the lesion epicenter (sections containing the least amount of spared tissue) and generated cell counts based upon P2y12 labeling of microglia and F4/80 labeling of MDMs. In the absence of injury, P2y12^+^ microglia were evident with a ramified phenotype (Fig. 3a). As reported previously, there was little to no staining for F4/80, the pan marker for activated macrophages, in uninjured tissue [17]. After SCI, F4/80^+^, round macrophages were clearly discernable at the lesion epicenter (Fig. 3). Microglia took on an amoeboid phenotype with P2y12 labeling punctate and concentrated in and around the nucleus as reported previously [17]. Using criteria, we established previously for SCI [8], we classified macrophages as MDMs (F4/80^+^ only) or microglia (F4/80^+^/P2y12^+^ or P2y12^+^ only).

Similar to flow cytometry, there were no main effects of sex or age in the density of total myeloid cells (F4/80^+^ macrophages) sampled (sex effect, *F*_(1, 23)_=0.0003, *p*=0.99; age effect, *F*_(1, 23)_=1.04, *p*=0.3). Interestingly, for MDM counts there were no main effects of sex (*F*_(1, 23)_=0.21; *p=*0.6) or age (*F*_(1, 23)_=0.41; *p=*0.5) indicating that MDM responses may be more widespread and that altered microglia numbers at the lesion epicenter may be driving the sex-dependent ratios observed using flow cytometry. Indeed, for microglia, we observed a significant main effect of sex (*F*_(1, 23)_=5.00; *p*<0.05) and a significant sex by age interaction (*F*_(1, 23)_=4.23; *p=*0.05) with a trend towards a main effect of age (*F*_(1, 23)_=3.68; *p*=0.07). Microglia counts were significantly increased in 4-MO males compared to 4-MO females (*p*<0.05) and 14-MO males (*p*<0.05). This sex-dependent increase in microglia is consistent with reports in TBI [9, 17] and may contribute, at least in part, to the significantly high MDM/microglia ratio found in females relative to males in our flow analysis.

### MDMs present with higher expression levels of activation- and ROS-associated genes

Having identified that sex significantly alters the inflammatory response to SCI at the level of cellular recruitment, we next examined the expression of phenotypic genes in MDMs and microglia as a function of both sex and age. MDMs and microglia were obtained from the injured spinal cords at 3-DPI and sorted into collection tubes using FACS based on gating strategies described above (Fig. 1a). A custom NanoString code-set was purchased to evaluate genes that were previously found to be specifically upregulated when activated using classical M1 (lipopolysaccharide and INFγ) and M2 (IL4) stimulation paradigms in vitro [12]. Due to obtaining low numbers of cells during sorting, the NanoString paradigm was not sensitive enough to reliably detect several genes in our code set (*n*=10); these genes were mainly interleukins and their receptors. All other genes that were reliably detected above the threshold were assessed (*n*=27; Table 1).

**Table 1:** Gene expression differences. RNA obtained from FACS sorted MDMs and microglia were compared between cell type, sex, and age. Estimated marginal means (EMM) were obtained following MANOVA to control for the combined variable when testing sex and age, and the EMMs were used for comparisons using *T* test (*p*) with, and without, false discovery rate (FDR) correction (*q*) using the two-stage step-up method of Benjamini, Krieger, and Yekutieli. Genes with fewer than 75% of samples testing below detectable threshold were not compared. Genes are organized based on affiliated function as previously described [12]. Significant *p* and *q* values were set to 0.05

Gene		Common names	Cell type		MDMs				Microglia			
					Sex		Age		Sex		Age	
			***p***	***q***	***p***	***q***	***p***	***q***	***p***	***q***	***p***	***q***
**M1 activation**	CD80	Cluster of differentiation 80	0.005*	0.001*	0.004*	0.057	0.788	1.000	0.049*	0.225	0.678	0.996
	CD86	Cluster of differentiation 86	0.001*	0.001*	0.526	0.897	0.059	0.981	0.534	0.726	0.904	1.000
	FCGR1	FC gamma receptor 1 (CD64)	0.005*	0.001*	0.087	0.396	0.855	1.000	0.936	0.940	0.830	0.996
	IL1b	Interleukin 1β	0.001*	0.001*	0.458	0.894	0.538	1.000	Below threshold		Below threshold	
	IL4Ra	Interleukin 4 receptor a	0.001*	0.001*	0.919	0.962	0.584	1.000	0.604	0.735	0.396	0.996
	TNF	Tumor necrosis factor	0.361	0.044*	0.370	0.894	0.562	1.000	0.866	0.909	0.252	0.996
	STAT1	Signal transducer and activator of transcription 1	0.253	0.032*	0.340	0.894	0.430	0.981	0.683	0.760	0.527	0.996
	SOCS3	Suppressor of cytokine signalling 3	0.472	0.055	0.812	0.962	0.983	1.000	0.987	0.950	0.423	0.996
**M2 activation**	STAT3	Signal transducer and activator of transcription 3	0.001*	0.001*	0.873	0.962	0.334	0.981	0.600	0.735	0.559	0.996
	ARG1	Arginase-1	0.001*	0.001*	0.876	0.962	0.369	0.981	0.025*	0.195	0.955	1.000
	CHI3L3	Chitinase 3-like-3	0.001*	0.001*	0.952	0.962	0.789	1.000	0.484	0.726	0.755	0.996
	MANF	Mes. astrocyte-derived neurotrophic factor	0.001*	0.001*	0.322	0.894	0.678	1.000	0.408	0.673	0.741	0.996
	MRC1	Mannose receptor C-type 1	0.001*	0.001*	0.698	0.908	0.267	0.981	0.003*	0.036*	0.637	0.996
	PPAR훾	Peroxisome proliferator activated receptor-훾	0.001*	0.001*	0.177	0.689	0.321	0.981	Below threshold		Below threshold	
	STAT6	Signal transducer and activator of transcription 6	0.001*	0.001*	0.041*	0.277	0.391	0.981	0.375	0.666	0.023*	0.308
	TGFβ1	Transforming growth factor β-1	0.001*	0.001*	0.052	0.283	0.323	0.981	0.069	0.264	0.362	0.996
**ROS**	SOD2	Superoxide dismutase 2	0.02*	0.003*	0.523	0.897	0.959	1.000	0.144	0.333	0.466	0.996
	CYBB	Cytochrome b-245 (NOX2)	0.001*	0.001*	0.696	0.908	0.450	0.981	0.003*	0.036*	0.024*	0.308
	EPAS1	Hypoxia-inducible factor 2-α	0.001*	0.001*	0.631	0.908	0.990	1.000	Below threshold		Below threshold	
	HIF1a	Hypoxia-inducible factor 1-α	0.001*	0.001*	0.015*	0.138	0.135	0.981	0.131	0.333	0.582	0.996
	HMOX1	Heme oxygenase-1	0.001*	0.001*	0.230	0.785	0.351	0.981	0.210	0.441	0.434	0.996
	IKBKB	Inhibitor of NFKB	0.001*	0.001*	0.904	0.962	0.450	0.981	0.100	0.329	0.780	0.996
	NFE212	Nuclear factor erythroid 2-related factor 2 (NRF2)	0.018*	0.003*	0.570	0.908	0.989	1.000	0.513	0.726	0.799	0.996
	SOD1	Superoxide dismutase 1	0.001*	0.001*	0.446	0.894	0.836	1.000	0.047*	0.225	0.513	0.996
	UCP2	Uncoupling protein 2	0.002*	0.001*	0.433	0.894	0.669	1.000	0.138	0.333	0.791	0.996
**Debris**	C1qa	Complement 1 subunit a	0.001*	0.001*	0.001*	0.018*	0.978	1.000	0.342	0.659	0.941	1.000
	cPLA2	Phospholipase A2	0.001*	0.001*	0.685	0.908	0.187	0.981	0.691	0.760	0.724	0.996

**Fig. 4:**
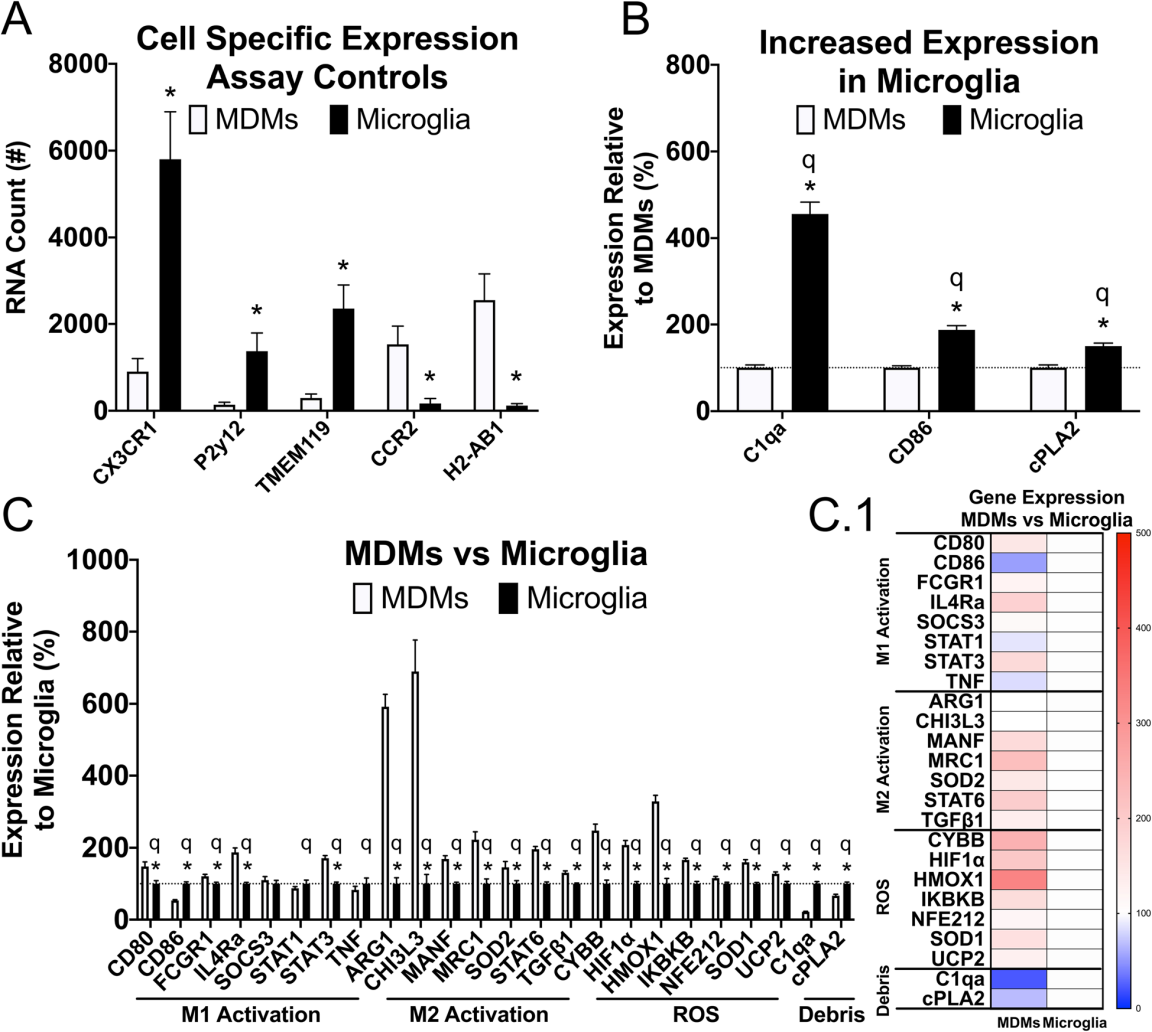
Activation-, ROS-, and debris-associated gene expression differs between MDMs and microglia at 3-DPI. MDMs and microglia were FACS sorted into separate tubes at 3-DPI, and RNA was isolated. RNA expression was quantified in up to 27 genes associated with activation, ROS, and debris response. **a** Genes known to be regulated specifically in MDMs or microglia were assessed to determine the purity and efficiency of FACS sorting and were analyzed using *T* test. All other analyses of RNA counts were assessed using a one-way MANOVA to determine the main effects between MDMs and microglia. Mean values were analyzed first by *T* test then by the two-stage step-up method of Benjamini, Krieger, and Yekutieli for FDR adjustment to control for multiple comparisons. **c** All but 3 genes were differentially regulated between cell types (*p*<0.05, C); SOCS3 (*p*=0.47), STAT1 (*p*=0.25), and TNF (*p*=0.36) were not different between MDMs and microglia. **b** All genes that were differentially regulated between cells were upregulated in MDMs with three exceptions; C1qa (*p*<0.001, *q*<0.001), CD86 (*p*<0.001, *q*<0.001), and cPLA2 (*p*<0.001, *q*<0.001) were upregulated in microglia. Sample size include MDMs (*n*=15) and microglia (*n*=14). All graphs present SEM. *Significant *T* test at least at *p*<0.05, ^q^significant discovery after FDR at least at *q*<0.05

First, RNA obtained from MDMs and microglia were compared to validate the sorting specificity. Samples from all sex and age groups were combined and a small subset of genes was separately analyzed as assay controls using *T* tests to determine the sensitivity of discriminating MDMs from microglia, as well as to validate our FACS sorting purity (Fig. 4a). CX3CR1 (*p*<0.001), P2y12 (*p*<0.001), and TMEM119 (*p*<0.001) were assessed as microglia-specific genes and were specifically expressed in our microglial populations. CCR2 (*p*<0.001) and H2-AB1 (*p*<0.001) were assessed as MDM-specific genes and were specifically expressed in our MDM populations. Results from these assay controls validate the FACS sorting success and demonstrates the sensitivity of discriminating between cell types using NanoString (Fig. 4a).

Since both MDM and microglia responses differed between males and females based upon our flow cytometry and histological results, we next examined the activation patterns between cell types. Samples from all sex and age groups were combined and used to compare the main effects between MDMs and microglia using MANOVA (Fig. 4c). Comparing main effects of cell type revealed a significant difference between MDMs and microglia in an overall expression of genes analyzed (*F*_(1, 27)_=388.02, *p*<0.05; Fig. 4c). Of the 27 genes assessed, after controlling for false discovery rate (FDR), all genes except SOCS3 (*q*=0.055) were deemed significant discoveries. Of the 26 genes that were significantly differentially expressed between MDMs and microglia, all but 3 genes were upregulated in MDMs (Fig. 4b). C1qa (*p*<0.001, *q*<0.001), CD86 (*p*<0.001, *q*<0.001), and cPLA2 (*p*<0.001, *q*<0.001) were significantly increased in microglia (Fig. 4b). This data suggests that within and surrounding the injured spinal cord at 3-DPI, MDMs are the primary expressors of ROS and conventionally defined M1 and M2 genes, while microglia are the major expressors of genes associated with a debris response. Since we observed sex-dependent MDM and microglia responses, these results implicate different secondary injury cascades in males and females after SCI.

### Being female after SCI increases activation- and ROS-associated genes while being male increases complement

**Fig. 5:**
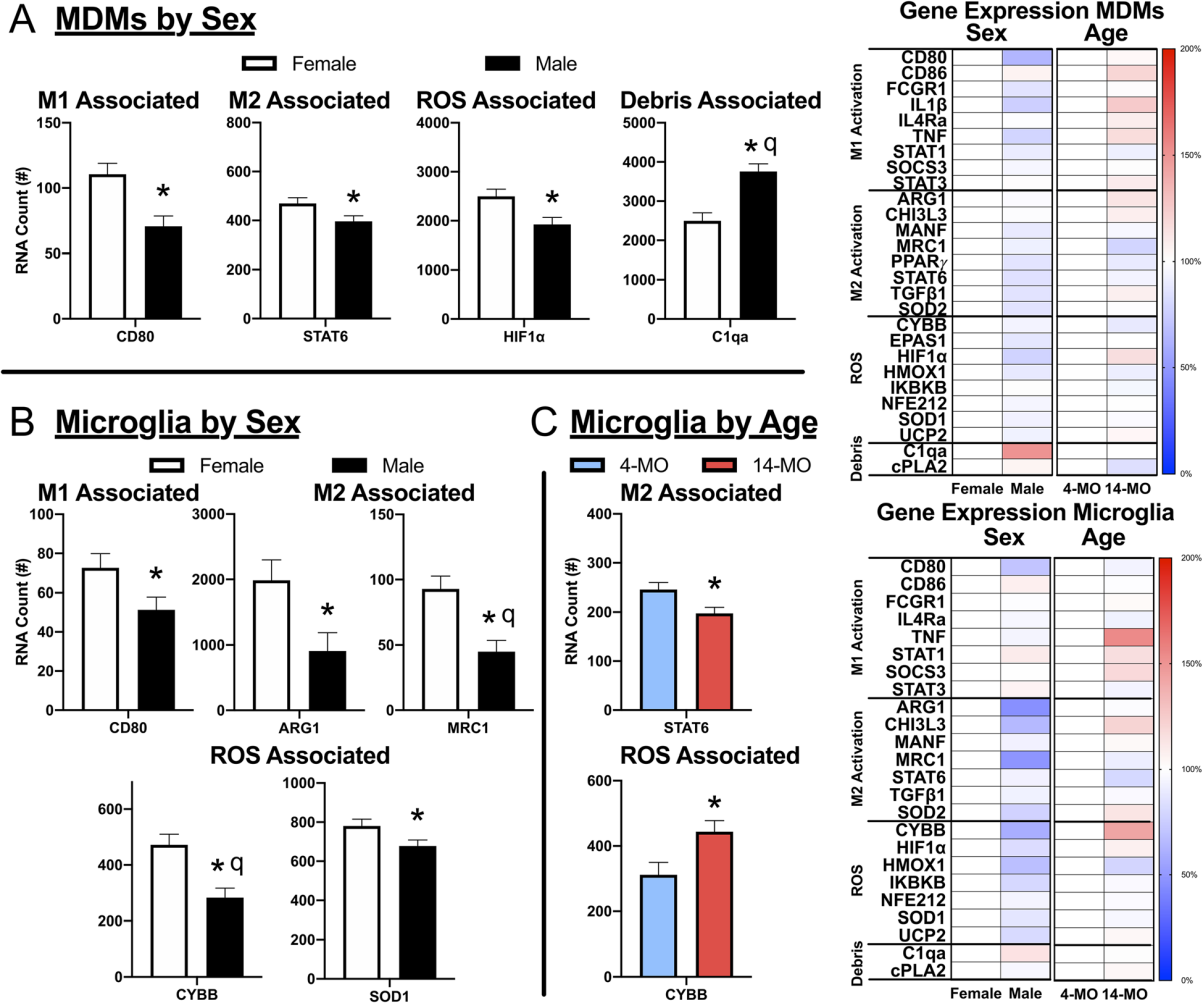
Activation, ROS, and debris-associated gene expression differs by sex in a cell-dependent manner at 3-DPI. MDMs and microglia were FACS sorted into separate tubes at 3-DPI, and RNA was isolated. RNA expression was quantified in up to 27 genes associated with activation, ROS, and debris response. RNA counts were assessed using a two-way MANOVA to determine the main effects between sex and age within each cell type. Because no interactions were found, the estimated marginal means with corresponding SEM’s were used to perform *T* tests and FDR analyses to correct for the combined variable. **a** A main effect of sex was found in MDMs (*p*<0.05), with 4 genes being significant at the level of *T* test, and only 1 gene persevering after FDR correction. **a** Specifically, CD80 (*p*<0.01*, q*=0.056), STAT6 (*p*<0.05*, q*=0.27), and HIF1α (*p*<0.05*, q*=0.13) were significantly increased in female MDMs, with C1qa (*p*<0.001, *q*<0.01) being upregulated in male MDMs as a significant discovery. No significant effects were found for age in MDMs. **b** Of 5 genes upregulated in female microglia, 2 were found to be significant discoveries. **b** Specifically, CD80 (*p*<0.05*, q*=0.22), Arg1 (*p*<0.05*, q*=0.19), and SOD1(*p*<0.05*, q*=0.22) were significantly increased in female microglia, with CYBB (*p*<0.01, *q*<0.05), and MRC1 (*p*<0.01, *q*<0.05), being significant discoveries after FDR correction. **c** Only two genes were significantly affected by age: STAT6 being decreased with age (*p*<0.05*, q*=0.30) and CYBB being increased with age (*p*<0.05*, q*=0.30). Sample size include females (MDMs, *n*=7; microglia, *n*=6), males (MDMs, *n*=8; microglia, *n*=8), 4-MO (MDMs, *n*=6; microglia, *n*=6), 14-MO (MDMs, *n*=9; microglia, *n*=8). All graphs present SEM. *Significant *T* test at least at *p*<0.05, ^q^significant discovery after FDR at least at *q*<0.05

Next, we examined whether the phenotype of MDMs and microglia differed by sex and age after SCI by examining differences in gene expression within each cell type. Individual two-way MANOVA’s were performed for each cell type to assess the main effects of sex and age, and the adjusted estimated marginal means (EMM) were used for pairwise comparisons. A FDR adjustment was performed on the EMMs obtained from each gene that were corrected for either sex or age. Analysis of MDMs revealed a significant main effect of sex (*F*_(1, 11)_=288.21, *p*<0.05; Fig. 5a), and significant sex-dependent differences in 4 of 27 genes analyzed with only 1 persevering through the FDR adjustment. CD80 (*p*<0.01), STAT6 (*p*<0.05), and HIF1α (*p*<0.05) were significantly upregulated in females, while C1qa was significantly upregulated in males both pre- and post-FDR correction (*p*<0.001, *q*<0.01; Fig. 5a). No genes were significantly increased between ages in MDMs (*F*_(1, 11)_=4.79, *p*<0.34).

While there was no main effect of either sex (*F*_(1, 10)_=77.75, *p*<0.08; Fig. 5b) or age (*F*_(1, 10)_=0.25, *p*<0.925; Fig. 5c) for microglia, there were 5 of 24 analyzed genes significantly upregulated in females, with 2 persevering through the FDR adjustment. Arg1 (*p*<0.05), CD80 (*p*<0.05), and SOD1 (*p*<0.05) were significantly upregulated in females, with both CYBB (*p*<0.01, *q*<0.05), and MRC1 (*p*<0.01, *q*<0.05) being significantly upregulated in females both pre- and post-FDR adjustment. CYBB (*p*<0.05) was also significantly upregulated in 14-MO microglia, while STAT6 (*p*<0.05) was significantly downregulated. Only 24 genes were analyzed in microglia due to a loss of three genes being below the threshold for detection; specifically, EPAS1, IL1β, and PPARγ were dropped from the analysis. Pairwise comparisons following MANOVAs of both MDMs and microglia did not reveal any significant interactions between sex and age for any genes. Collectively, gene expression analyses indicate greater activation states in MDMs than microglia. Together, these analyses identify complement as potentially being disproportionally activated in males due to a higher gene expression in MDMs as well as the presence of more complement carrying microglia. In contrast, females have a larger proportion of ROS-producing MDMs with their microglia expressing more ROS-associated genes.

## Discussion

Data collected from our experiments support that inflammation after SCI is sex-dependent both at the level of cellular recruitment and phenotype. Effects of aging, while present, were less pronounced in microglial gene expression. We found that MDM/microglia ratios throughout the injured spinal cord of female mice were larger, which may be partially explained by 4-MO male mice having more microglia at the lesion epicenter. Further, transcriptional profiles of MDMs and microglia indicate that female macrophages are more robustly activated after SCI and being both female and older increase ROS-producing genes in microglia. In contrast, male mice exhibit a unique increase in C1qa expression in MDMs with a concurrent increase in microglial density at the lesion epicenter. Collectively, these data indicate that inflammatory profiles are sex-dependent after SCI and that mechanisms of secondary injury may diverge towards females having more ROS-, and males having more complement-driven pathology.

There is increasing evidence that the pathophysiology and recovery after SCI may be sex-dependent [13, 14, 18–23]. This is especially true for neuropathic pain where sex-dependent outcomes have been observed in both humans and rodents [24–26]. Further, we have recently observed that the effectiveness of immunomodulatory analgesics is sex-dependent [27]. Pain development post-SCI is associated with inflammation and inflammatory cascades within the SCI lesion and lumbar spinal cord [28–30]. Here, we demonstrate that inflammatory recruitment in response to SCI is indeed sex-dependent, which provides a foundation to begin understanding sex differences in neuropathology and post-SCI complications, such as pain, that arise after injury.

In addition to sex differences in cellular recruitment, we observed important differences in gene profiles of two important pathways involved in secondary injury cascades. Specifically, females had an increase in activation, as well as ROS-associated genes, while males had an increase in C1qa of the complement pathway. Previously, we have reported in data from only female mice, that age increases NOX2 and ROS damage, which worsens function after SCI [8]. NOX2 may play a larger role in SCI pathophysiology in female mice as, in the current study, we detected that NOX2 was increased in female microglia compared to males, which was further increased with age. Our observation of increased CYBB expression (gene encoding for NOX2) with age is consistent with prior studies conducted in females [7, 31]. Male-dependent increases in C1qa in MDMs after SCI has not been reported previously. Not only was C1qa significantly increased in male MDMs, but males also possessed more microglia within the SCI lesion, of which microglia are the primary native producers of complement in the nervous system. Indeed, we detected a roughly five-fold increase in C1qa gene expression in microglia compared to MDMs. These data collectively point to potentially meaningful differences driving sex-dependent pathology after SCI.

It remains important to consider that even if differences in functional outcomes are subtle between sexes or age after SCI [14], that the molecular and biological responses can be considerably different [13]; our data supports this concept. Implications for such discrepancies in biological responses are that treatments tailored to any given mechanism may also exert sex-dependent or age-dependent effects [32]. For example, previous work from our lab has used pioglitazone to treat SCI pain and found a significant analgesic effect in female, but not male, mice [27]. While the mechanisms underlying this sex-dependent analgesic effect are unknown, there have been reports of estrogen directly influencing pioglitazone biological target, PPARγ [33–35], as well as reports of the increased potency of pioglitazone on insulin sensitization in females [36]. Here, we have found that NOX2 and C1qa may play larger contributors to SCI pathology in female and male mice, respectively. Further, we observed that MDM/microglia ratios are significantly affected by age. Considering our prior work demonstrating age-dependent and age-divergent therapeutic efficacy of NOX2 inhibition and other therapies targeting ROS in female mice [8, 37], future work should determine if a similar effect is shared in males.

As part of the complement cascade, C1qa plays an important role in responding to cellular debris after injury and apoptosis [38–40]. However, C1qa after SCI is also a known contributor to secondary injury cascades [39, 41, 42]. When C1qa is globally knocked out or suppressed, mice perform better on locomotor tasks and lesion severity is reduced [41]. While the role of C1qa as a sex-dependent exacerbator of SCI has not yet been examined, both age and sex are known to differentially affect complement activation in the blood of healthy adult humans [43] and mice [41]. Our findings have specifically identified a male-dependent increase in C1qa within infiltrating monocytes after SCI, with a further male-dependent increase in microglial counts, which are the largest producers of C1qa within the spinal cord. Owing to the strong contribution of complement to SCI pathology, future work should evaluate potential sex differences in targeting complement activation as a therapy.

Male- and female-associated sex hormones differentially affect inflammation in response to injury or infection; therefore, similar sex-dependent inflammatory responses to neurotrauma are likely [44–48]. Indeed, differences in acute inflammatory profiles between sexes exist following brain injury, specifically finding an increase in microglia and total inflammation in male rodents at 3-DPI [9, 17]. This finding is consistent with suggestions that testosterone may enhance microglial activation but is inconsistent with reports that testosterone suppresses MDMs [48, 49]. In contrast, estrogens have been found to increase MDM activation and suppress microglia [44, 45, 47].

Increased age at the time of SCI also affects inflammation by both increasing leukocyte infiltration as well as changing genetic expression of activation- and ROS-associated genes [6, 7, 31]. Our lab has found that advanced age at the time of SCI increases total inflammation by 3-DPI and shifts macrophage phenotypes towards a more aggressive ROS-producing profile [7, 8]. This increase in ROS damage with age corresponds with an increase in macrophage-specific NOX2 expression, which can be mitigated in an age-dependent manner using apocynin as a NOX2 inhibitor [8]. Apocynin reduced ROS production in a cell-specific manner, specifically reducing MDM-derived ROS [8]. Data presented in this study further corroborates the role of age on increasing NOX2 expression and identified microglia as the macrophage cell-type that is most affected by aging.

It is important to consider technical differences in flow cytometry and immunohistochemistry methods of analysis that can partially explain discrepancies between these data. First, when extracting spinal cords for flow cytometry, a consistent length of cord was removed. Overall cell numbers did not differ among groups making it unlikely that an increased spinal cord size reported with advanced age influenced our results [50]. We determined cell counts through flow cytometry without normalization to total tissue or lesion size. Indeed, our observation of increased MDMs/microglia ratios in 4- vs. 14-MO females is consistent with previous histological examination when total cell numbers were counted without normalization to tissue area [8]. Since we previously observed that the rostral-caudal length of the lesion increases in males vs. females and in 14- vs. 4-MO mice [6, 14], we only examined the lesion epicenter and normalized to tissue area for histological cell counts. It is not surprising, therefore, that we did not make the same observations with histological and flow analyses. Indeed, our histological analyses revealed a consistent MDM density between groups; however, we detected a significant increase in microglial density within the lesion of 4-MO males. Immune cells within the lesion core are predominately MDMs, making our observation of increased microglial responses in males intriguing, and collectively, we conclude that the profile of inflammation after SCI is sex-dependent.

Our findings of increased activation- and ROS-associated genes in MDMs relative to microglia should be interpreted with caution. When extracting myeloid cells from the injured spinal cords a region of tissue is isolated that is slightly larger than the immediate lesioned environment. This will result in isolating microglia at distances away from the lesion that may have not yet been recruited or activated from injury. In contrast, all infiltrating MDMs are likely activated through the migratory process, which would result in a larger percentage of the MDM population expressing activation genes. Our data comparing mRNA from MDMs vs microglia may simply reflect the fact that isolated mRNA is derived from a fraction of activated microglia, whereas likely all MDM mRNA came from activated cells. Or, alternatively, the association between classically defined M1 and M2 genes obtained from in vitro paradigms, as previously reported [12], may indeed be a better representation of gene expression profiles of activated MDMs compared to microglia. Nevertheless, microglia did persist to demonstrate an increased expression of the two genes, C1qa and CPLA2, which may underly critical functional differences in cellular and sex-dependent responses after SCI.

## Conclusions

Collective data, presented here, confirm a sex-dependent inflammatory effect at both the level of cell infiltration as well as gene expression profile. Consistent with reports in TBI [9], we have found an increased density of microglia in male mice, with a corresponding increase in the proportion of monocytes in female mice, after SCI. Further, we have validated that the gene transcript encoding complement protein C1qa is indeed increased in males [41], specifically finding an increased expression in male MDMs. In addition to male-dependent complement activation, our findings indicate that the response of microglia and monocyte-derived macrophages are both age and sex-dependent after SCI with increased NOX2 expression in microglia from female and older animals. Findings from this study highlight the importance of considering both age and sex as important biological variables in the pathophysiology and immunomodulatory treatment of spinal cord injury.

## Supplementary Information

**Additional file 1.**

**Additional file2: Supplementary Figure 1**. Annotation markup for MDM and microglial cell counts. Unbiased sampling procedures were used to count MDMs and microglia throughout the lesion. **a** Lesions were traced (blue trace) and partitioned into 100 μm^2^, randomly placed, boxes that were evenly spaced at 100 μm apart (green boxes). **b** Within each sampling area, MDMs (brown cells; red circles) were counted as long as cresyl violet nuclei were contained within, or touching, the right or top margins of the box. Microglia (black cells; blue measuring bar; blue arrow) were counted that met pre-defined criterion of presenting with a soma and at least 5 μm in length or diameter, and were contained within, or touching, the right or top margins of the box. Cells with nuclei touching the bottom or left margins (yellow arrow) were excluded from cell counts.

## Data Availability

Data generated in this study is available upon request and will be deposited in the Open Data Commons for Spinal Cord Injury upon publication (ODC-SCI; https://scicrunch.org/odc-sci).

## Abbreviations

ANOVA: Analysis of variance
DAB: 3,3’-Diaminobenzidine
DPI: Day post-injury
EDTA: Ethylenediaminetetraacetic acid
EMM: Estimated marginal means
F4/80: Glycoprotein found on all macrophages
FACS: Fluorescence-activated cell sorting
FDR: False discovery rate
MANOVA: Multiple analysis of variance
MDM: Monocyte-derived macrophage
MO: Month old
NOX2: NADPH oxidase
P2y12: Chemoreceptor for ADP found on microglia but not MDMs
PBS: Phosphate-buffered saline
ROS: Reactive oxygen species
SCI: Spinal cord injury
TBI: Traumatic brain injury
