# Supplementary figures and images for: Acute inflammatory profiles differ with sex and age after spinal cord injury
Source: J Neuroinflammation. 2021 May 13;18:113. doi: 10.1186/s12974-021-02161-8 (PMC8120918; doi:10.1186/s12974-021-02161-8)

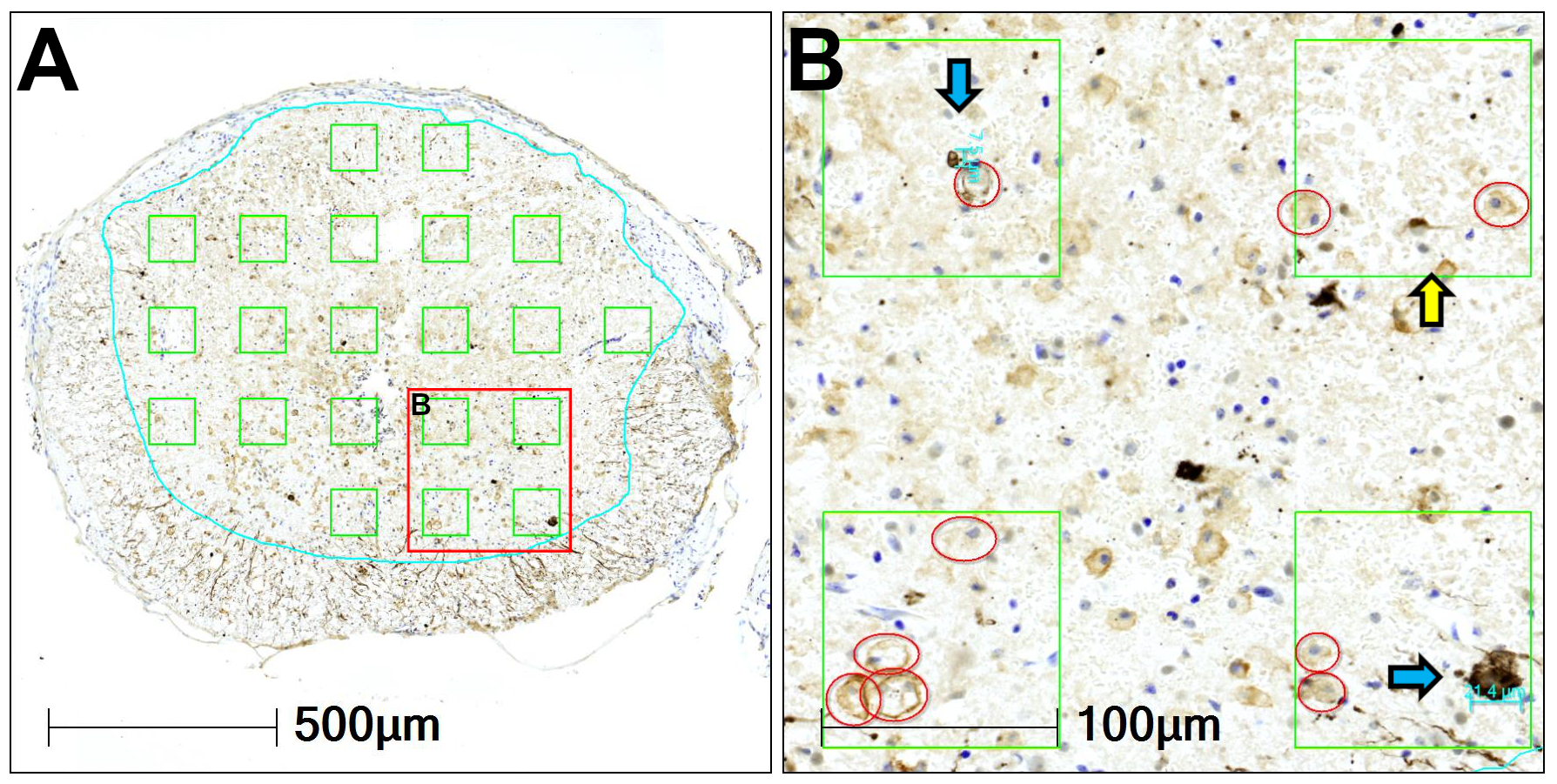

Supplement: Supplementary file 2 — Additional file2: Supplementary Figure 1. Annotation markup for MDM and microglial cell counts. Unbiased sampling procedures were used to count MDMs and microglia throughout the lesion. a Lesions were traced (blue trace) and partitioned into 100 μm2, randomly placed, boxes that were evenly spaced at 100 μm apart (green boxes). b Within each sampling area, MDMs (brown cells; red circles) were counted as long as cresyl violet nuclei were contained within, or touching, the right or top margins of the box. Microglia (black cells; blue measuring bar; blue arrow) were counted that met pre-defined criterion of presenting with a soma and at least 5 μm in length or diameter, and were contained within, or touching, the right or top margins of the box. Cells with nuclei touching the bottom or left margins (yellow arrow) were excluded from cell counts. [file 12974_2021_2161_MOESM2_ESM.jpg]
